# Georgia Medical Association

**Published:** 1875-06

**Authors:** 


*Georgia Medical Association.*

This body held its annual session in Savannah Georgia, April last. While it was not our good fortune to be present, we understand the meeting was well attended, and one of more than usual interest in matters touching the welfare of the profession in the State.

Dr. J. G. Thomas, of Savannah, was elected President.

The next annual meeting will be held in Augusta, Georgia.

Before closing we desire to extend our thanks for the complimentary resolution offered the RECORD, and our best wishes for the happiness and continued prosperity of every worthy member.

